# Observations on a Recent Publication on the Structure of the Parts Concerned in Crural Hernia

**Published:** 1806-08

**Authors:** Allan Burns

**Affiliations:** Lecturer on Anatomy and Surgery at Glasgow


*Observations on a recent Publication on the Structure of the Parts concerned in Crural Hernia, by Mr. Allan Burns, Lecturer on Anatomy and Surgery at Glasgow.*

THE attention of Surgeons has been in a particular manner directed to this subject, since the publication of Mr. Hey's Practical Observations in Surgery, in which that valuable writer brings into notice the accurate Essay of Don Antonio de Gimbernat. Every description, however, of the Anatomy of the Groin, which has of late years appeared in the writings of Surgeons, although no doubt calculated to extend, in some degree, our acquaintance with the structure of this part, have, in our opinion, been more or less imperfect. We were hopeful, therefore, that Mr. Burns, in his publication, would have supplied any deficiencies connected with this subject, and described such peculiarities as have remained unnoticed by other Anatomists. In these expectations, however, we have been extremely disappointed; there is nothing in Mr. Burn's Essay which is in the least interesting; nothing which can with any propriety be considered as new or original. We willingly allow him the merit of having described with great minuteness, that reflexion of Poupart's ligament which lines the anterior surface of the iliacus internus and psoas muscles, as well that which is

 expanded expanded over the posterior surface of the transversalis abdominis; but at the same time we must be permitted to observe, that the description of the former by Gimbernat, and of the latter by Mr. Cooper, is fully as accurate, and much more intelligible. We are rather inclined to suspect, that Mr. Cooper's work on Hernia has not yet fallen into the author's hands: if so, we beg leave to recommend that gentleman's book to Mr. Burn's early perusal. But if our suspicions are groundless, we do not hesitate to say, that Mr. Cooper's name ought not, in this communication, to have been passed over in such total silence. We could have wished that Mr. Burns had directed less of his attention to the internal reflexions from Poupart's ligament, and dissected with greater accuracy those external parts which are of more immediate importance in the operation for crural hernia. His description of the fascia lata of the thigh is, we apprehend, much more calculated to perplex than to instruct. According to him, this fascia consists of two layers, the outer of which is thin and cellular, the inner one firmer, thicker, and more membranous. Now this outer, thin, and cellular layer, appears to us to be neither more nor less than the loose fascia in which the inguinal glands are usually embedded, and which was first accurately described and delineated by Camper. The inner and firmer layer is the fascia lata of the thigh, properly so called, and the curved, the lunated, the crescentic margin of this fascia, the falciform process so minutely described by Mr. Burns, is exactly the femoral ligament of Mr. Hey. Here, indeed, the correspondence of description is so striking, that we were sorry to find no candid acknowledgement on the part of Mr. Burns, of the many and evident obligations which he lies under to pages 151, 152, 153, &c. of Mr. Hey's Practical Observations.

That the femoral ligament described by Mr. Hey does actually exist, we are fully convinced from repeated dissections. "It may be easily discovered, by introducing the finger (after the abdomen has been laid open) into the aponeurotic sheath of the great femoral vessels, behind Poupart's ligament. As the finger passes downwards it will be pressed upon by a part of the fascia, more compact than the rest, which runs transversely about three-eighths of an inch below Poupart's ligament, and sometimes presents a sharp edge to the finger. It is not situated in the same plane with the ligament of Poupart, but lies lies deeper, that is, at a greater distance from the integuments." (Practical Observations, p. 152, 154.)

So far this is certainly correct; but we apprehend that Mr. Hey has fallen into an error, when he says, (p. 153) that "this femoral ligament is formed in the fascia of the thigh, or the anterior layer of the aponeurotic sheath of the great femoral vessels." He does not seem to be aware that the anterior layer of the sheath of the vessels is formed by a distinct reflexion downwards from Poupart's ligament, and not by the fascia lata of the thigh, between which and the reflexion we have mentioned, there is in most subjects a distinct layer of fat and cellular substance. It is the curved margin of this reflexion which is felt in the situation pointed out by Mr. Hey, pressing upon the finger when introduced from the abdomen into the crural ring; and we believe that, upon careful examination, it will be found to be this same reflexion, and not the falciform process of the fascia lata, as Mr. Burns asserts, which is continued into the ligament of Gimbernat. By dividing, therefore, a few of the fibres at the margin of this reflexion, a stricture existing in the crural ring would be as effectually removed as by Gimbernat's operation.

That Mr. Burns may be fully satisfied of the justice of our remarks, we have only to request that he will repeat with care his dissections of the groin. On cutting through the skin and cellular membrane, Mr. Burns will find, that the first part which presents itself is the loose fascia of Camper. This being removed, the fascia lata of the thigh is brought into view. Let now the fascia lata be carefully lifted up, and another facia will be discovered, a reflexion sent off from the middle and internal edge of Poupart's ligament, which running downwards over the femoral artery and vein, constitutes the anterior sheath of the femoral vessels. Between this fascia and the fascia lata there is in most subjects a layer of fat, nearly a quarter of an inch in thickness. In some cases there is only a thin layer of cellular substance interposed, but in every instance the fascia are sufficiently distinct to admit of separate demonstration.

In our dissections of these parts, we have uniformly followed the very accurate description of them, which we have found in Notes from the Lectures of Mr. Thomson, Professor of Surgery in Edinburgh.

